# Muse+TriposScore: a ligand-based de novo design approach

**DOI:** 10.1186/1758-2946-3-S1-P26

**Published:** 2011-04-19

**Authors:** F Bös, U Uhrig, BB Masek, JR Damewood

**Affiliations:** 1 Tripos International, Martin-Kollar-Straße 17, 81829 München, Germany; 2 Tripos International, 1699 South Hanley Road, St. Louis, Missouri 63144, USA; 3 AstraZeneca Pharmaceuticals, 1800 Concord Pike, Wilmington, Delaware 19850, USA

## 

Successful drug discovery often requires optimization against a set of biological and physical properties. We describe our work on multi-parameter approaches to ligand-based de novo design and studies that demonstrate its ability to successfully generate lead hops or scaffold hops between known classes of ligands for some example receptors. We describe a multi-criteria scoring function incorporating molecular shape similarity, molecular fingerprint similarity, and a number of popular “Lipinski-like” molecular properties.

Muse is based on an evolutionary algorithm that operates on an initial population of structures to invent new structures with improved scores. Muse is unique in that it has the ability to work with any user-defined scoring function that provides results in the form of a numerical evaluation of designed structures.

Several retrospective studies on various targets demonstrate the ability of the above mentioned approach to generate novel ideas that are not only appealing to design scientists but are also validated by comparison to compounds known to demonstrate activity at the desired biological target. Specific examples where this approach has generated either significant modifications of existing molecular frameworks or structurally new molecular templates relative to design starting points (i.e., lead hopping) will be provided.

